# Sex-Dependent T Cell Dysregulation in Mice with Diet-Induced Obesity

**DOI:** 10.3390/ijms25158234

**Published:** 2024-07-28

**Authors:** Christina Brummer, Katrin Singer, Almut Brand, Christina Bruss, Kathrin Renner, Wolfgang Herr, Tobias Pukrop, Christoph Dorn, Claus Hellerbrand, Carina Matos, Marina Kreutz

**Affiliations:** 1 Department of Internal Medicine III, Hematology and Oncology, University Hospital Regensburg, Franz-Josef-Strauß Allee 11, 93053 Regensburg, Germany; 2 Bavarian Cancer Research Centre (BZKF), 93053 Regensburg, Germany; 3 Department of Gynecology and Obstetrics, University Hospital Regensburg, Franz-Josef-Strauß Allee 11, 93053 Regensburg, Germany; 4 Department of Otorhinolaryngology, University Hospital Regensburg, Franz-Josef-Strauß Allee 11, 93053 Regensburg, Germany; 5 Comprehensive Cancer Center Eastern Bavaria (CCCO), 93053 Regensburg, Germany; 6 Center of Translational Oncology (CTO), 93053 Regensburg, Germany; 7 Institute of Pharmacy, University of Regensburg, 93053 Regensburg, Germany; 8 Institute of Biochemistry, University of Erlangen, 91054 Erlangen, Germany

**Keywords:** obesity, gender, sex, inflammation, immune cell dysregulation, T cell, MDSC, female, male, cancer

## Abstract

Obesity is an emerging public health problem. Chronic low-grade inflammation is considered a major promotor of obesity-induced secondary diseases such as cardiovascular and fatty liver disease, type 2 diabetes mellitus, and several cancer entities. Most preliminary studies on obesity-induced immune responses have been conducted in male rodents. Sex-specific differences between men and women in obesity-induced immune dysregulation have not yet been fully outlined but are highly relevant to optimizing prevention strategies for overweight-associated complications. In this study, we fed C57BL/6 female vs. male mice with either standard chow or an obesity-inducing diet (OD). Blood and spleen immune cells were isolated and analyzed by flow cytometry. Lean control mice showed no sex bias in systemic and splenic immune cell composition, whereas the immune responses to obesity were significantly distinct between female and male mice. While immune cell alterations in male OD mice were characterized by a significant reduction in T cells and an increase in myeloid-derived suppressor cells (MDSC), female OD mice displayed preserved T cell numbers. The sex-dependent differences in obesity-induced T cell dysregulation were associated with varying susceptibility to body weight gain and fatty liver disease: Male mice showed significantly more hepatic inflammation and histopathological stigmata of fatty liver in comparison to female OD mice. Our findings indicate that sex impacts susceptibility to obesity-induced T cell dysregulation, which might explain sex-dependent different incidences in the development of obesity-associated secondary diseases. These results provide novel insights into the understanding of obesity-induced chronic inflammation from a sex-specific perspective. Given that most nutrition, exercise, and therapeutic recommendations for the prevention of obesity-associated comorbidities do not differentiate between men and women, the data of this study are clinically relevant and should be taken into consideration in future trials and treatment strategies.

## 1. Introduction

Overweight affects more than one-third of the world’s population and is associated with increased morbidity and mortality [1]. Due to a sedentary lifestyle and calorie-rich, highly processed food, the numbers of obese people in industrial countries are constantly rising [2]. Obesity-associated secondary diseases such as metabolic-associated fatty liver disease (MAFLD), diabetes mellitus type 2 (T2DM), cardiovascular disease (CVD), and several types of cancer account for most deaths worldwide [3]. Thus, the World Health Organization (WHO) already considered obesity and its comorbidities an emerging public health problem years ago [4].

To prevent obesity-induced complications, understanding the underlying pathophysiological mechanisms is mandatory [5]. The current literature proclaims the development of obesity-associated secondary diseases to be multifactorial with numerous contributing parameters including genetic predisposition, dietary habits, altered gut microbiome, and systemic low-grade inflammation [6,7]. The interplay between obesity-induced immunologic and metabolic dysregulation has been the subject of extensive research in recent decades [8,9]. It is commonly known that obesity-induced lipid accumulation leads to the dysfunction of adipocytes and loss of local immune regulation [10]. Imbalanced adipose tissue (AT) immune homeostasis results in the increased release of hormones and pro-inflammatory cytokines such as the C reactive protein (CRP), interleukin (IL) 6, IL18, and tumor necrosis factor (TNF), as well as the impaired function of circulating immune cells promoting chronic systemic low-grade inflammation [9,11]. Consequently, obesity has been shown to lead to the dysregulation of the circulating T cell compartment [12,13]. In line with this, obesity is associated with impaired immune responses to infections and vaccinations [14,15].

Systemic immune dysregulation is regarded as one major driver for the development of obesity-induced secondary diseases [16]. In recent years, there has been rising evidence that men and women differ in their risk of developing obesity-associated complications [17,18,19]. For example, men are at higher risk of developing CVD and T2DM at a younger age and lower body mass index (BMI) than women [17,18,19]. Vice versa, women have been found to gain more weight than men when first diagnosed with T2DM [17,18]. However, most preliminary studies about obesity-induced immune cell alterations have focused on male study participants, both in rodents and humans [20]. Data comparing male vs. female susceptibility to obesity-induced inflammation are rare, and differences have not yet been fully elucidated [20].

In this study, we analyzed the blood, spleen, and livers of male vs. female C57BL/6 mice fed either standard chow or an obesity-inducing diet (OD). While in OD-fed male mice, the reprogramming of circulating and splenic immune cells was characterized by a significant reduction in T cells and the concomitant expansion of myeloid-derived suppressor cells (MDSC), female OD mice displayed preserved systemic T cell numbers. The sex-dependent difference in obesity-induced systemic T cell dysregulation was associated with distinct susceptibility to body weight gain, hepatic inflammation, and the severity of fatty liver disease. Our findings indicate that sex might impact the sensitivity to chronic obesity-induced systemic inflammation and therefore the incidence of obesity-induced secondary diseases.

## 2. Results

### 2.1. Male Mice Are More Susceptible to Diet-Induced Obesity and Fatty Liver Disease than Female

**Figure 1:**
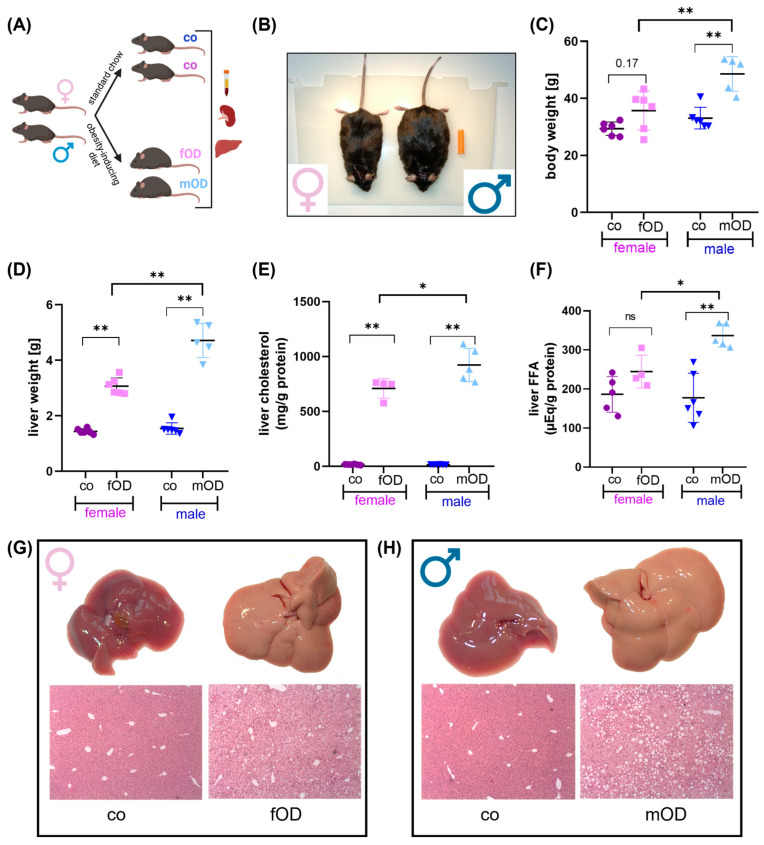
Male mice are more susceptible to diet-induced obesity and fatty liver disease than females. (**A**) Experimental design: Seven-week-old female (pink) or male (blue) C57BL/6 mice were fed standard chow (co) or an obesity-inducing diet (OD) for 38 weeks. At 45 weeks of age, blood, spleens, and livers were analyzed. (**B**) Phenotype comparison between female (left) and male (right) mice with diet-induced obesity at 45 weeks of age. (**C**) Body weight of female and male control vs. OD mice at week 45. (**D**) Liver weight at 45 weeks. (**E**,**F**) Liver cholesterol (**E**) and free fatty acid (FFA, **F**) content of female and male control vs. OD mice at week 45. (**G**,**H**) Pictures and HE staining of livers of female (**G**) vs. male (**H**) mice at week 45. Microscopic images were obtained at 40× magnification. Significance is tested by Mann–Whitney U test and indicated for *p* < 0.05 (*) and *p* < 0.01 (**).

To characterize sex differences in the onset of obesity and obesity-associated secondary diseases, seven-week-old male and female C57BL/6 mice were fed standard chow (control) or an obesity-inducing diet (OD) mimicking the nutrition habits in modern societies (Figure 1A). After 30 weeks of the diet, mice were analyzed in terms of their phenotype, body and liver weight, histopathological stigmata of fatty liver disease, and serum and hepatic metabolic parameters.

At week 45, both female and male OD mice could be differentiated from their lean littermates by phenotype change and body weight gain (Figure 1B,C). However, despite equal food consumption (Supplementary Figure S1A), male mice developed obesity at a younger age and in a more pronounced manner than females (Figure 1B,C). While female OD (fOD) mice differed in their body weight from control mice no earlier than week 30 (Figure 1B, Supplementary Figure S1B), OD male (mOD) mice already started to gain more body weight than lean control mice at week 20 (Figure 1C, Supplementary Figure S1C).

The obese phenotype of both fOD and mOD mice was associated with hypercholesterinemia (Supplementary Figure S1D), increased liver weight (Figure 1D), hepatic triglyceride (Supplementary Figure S1E), cholesterol (Figure 1E), and free fatty acid content (Figure 1F). Thereby, liver weight gain and the accumulation of hepatic lipid content were significantly more pronounced in males than females (Figure 1D–F). Macroscopical and microscopical analyses of livers (Figure 1G,H) confirmed that mOD mice revealed more severe fatty liver disease in comparison to their female littermates (fOD). In line with this, serum levels of alanine amino transferase (ALAT), as a systemic marker for liver cell damage, were higher in mOD than in fOD mice (Supplementary Figure S1F).

Taken together, in this diet-induced obesity mouse model, male mice displayed a more pronounced obese phenotype earlier than their female littermates, which has been described before [17,18,19]. In line with this, male mice were more susceptible to metabolic-associated fatty liver disease (MAFLD).

### 2.2. Obesity-Induced Liver Inflammation Is More Pronounced in Males than Females

**Figure 2:**
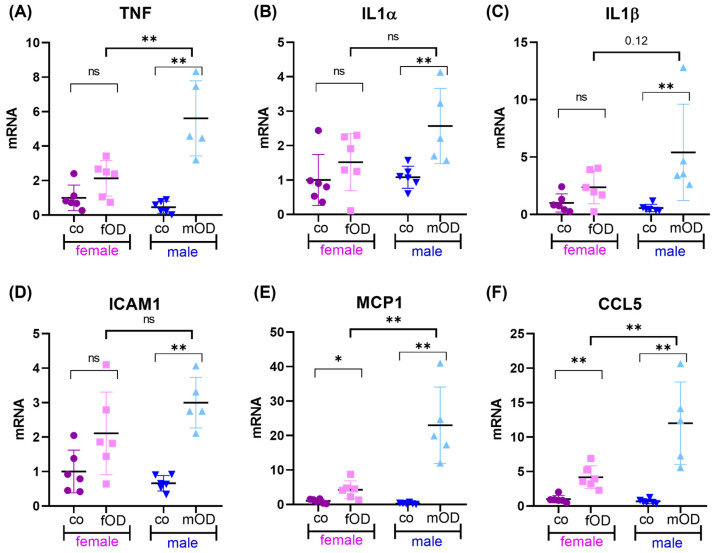
Obesity-induced liver inflammation is more pronounced in males than females. Female (pink) and male (blue) C57BL/6 mice were fed standard chow (co) or an obesity-inducing diet (OD) for 38 weeks. At 45 weeks of age, livers were analyzed in terms of pro-inflammatory response. Relative mRNA expression of (**A**) TNF, (**B**) IL1α, (**C**) IL1β, (**D**) ICAM1, (**E**) MCP1, and (**F**) CCL5 is shown normalized to 18S RNA. Significance is tested by Mann–Whitney U test and indicated for *p* < 0.05 (*) and *p* < 0.01 (**). Non-significant results are labelled ns.

The imbalance between anti- and pro-inflammatory homeostasis towards chronic inflammation is known to contribute to the development of obesity-induced secondary diseases and indicates the progression from simple fatty liver to steatohepatitis. To further evaluate whether sex-dependent distinct susceptibility to MAFLD was associated with differences in hepatic inflammation, we next analyzed the liver tissue of fOD vs. mOD mice in terms of the pro-inflammatory response (Figure 2).

Hepatic qPCR analysis revealed that both female and male OD mice exhibited significantly increased mRNA expression of pro-inflammatory mediators such as tumor necrosis factor (TNF, Figure 2A), interleukin 1α (IL1α, Figure 2B), interleukin 1β (IL1β, Figure 2C), intercellular adhesion molecule 1 (ICAM1, Figure 2D), monocyte chemoattractant protein 1 (MCP1, Figure 2E), and chemokine ligand 5 (CCL5, Figure 2F) in comparison to lean control mice. In line with the results of histopathological analyses, the pro-inflammatory response regarding TNF, MCP1, and CCL5 was significantly more pronounced in male than in female OD mice (Figure 2A,E,F). A similar but non-significant trend could be observed for IL1α, IL1β, and ICAM1 (Figure 2B–D).

In summary, these results indicate that male mice are more susceptible to not only the development of fatty liver disease but also its progression toward steatohepatitis.

### 2.3. Obesity-Induced Systemic Immune Cell Alterations in Males Are Only Partly Reflected in Females

**Figure 3:**
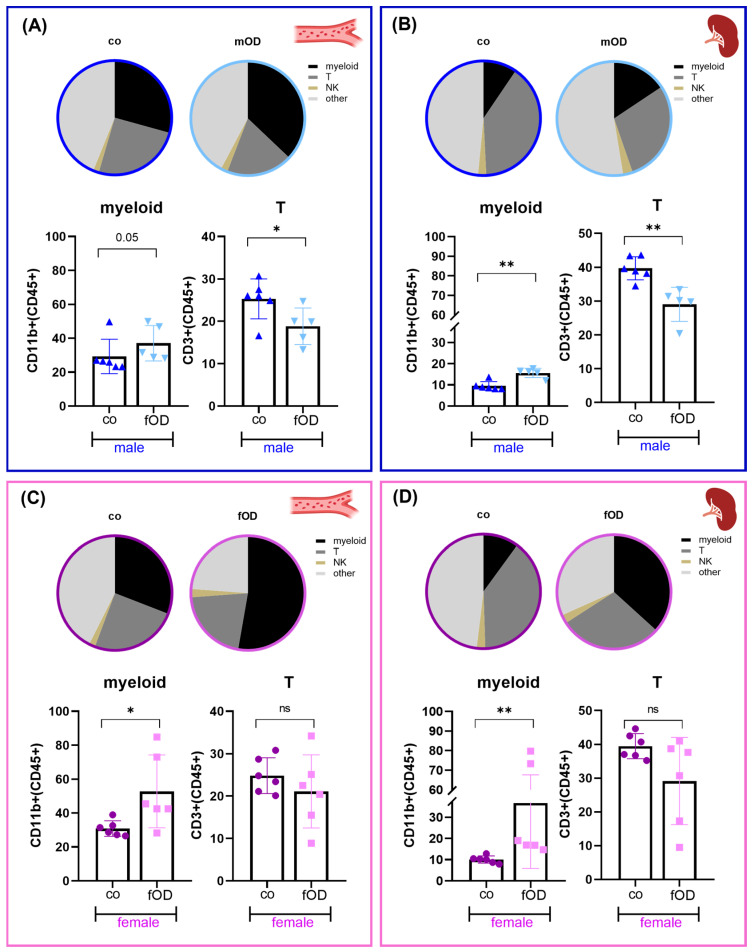
Male-typical obesity-induced systemic immune cell alterations in the myeloid and T cell compartment are only partly reflected in females. Seven-week-old male (blue, **A**,**B**) and female (pink, **C**,**D**) mice were fed standard chow (co) or an obesity-inducing diet (OD). At 45 weeks, blood (**A**,**C**) and spleen (**B**,**D**) immune cells were analyzed by flow cytometry. Cell subtypes are shown as fractions of total CD45+ cells. Results represent the mean ± SD of n = 5–6 mice per group. Differences were tested by Mann–Whitney U test. Significance is indicated for *p* < 0.05 (*) and *p* < 0.01 (**). Non-significant results are labelled ns.

Obesity is known to induce not only adipose tissue and liver inflammation but also systemic inflammation, which is regarded as a hallmark for the development of obesity-associated secondary diseases. To evaluate whether the observed different susceptibility to fatty liver disease in female and male OD mice is associated with distinct systemic immune cell dysregulation, we next analyzed the blood and spleen leukocyte composition of lean control vs. OD female (pink) and male (blue) mice via flow cytometry (Figure 3).

Lean control mice exerted similar circulating and splenic immune cell compositions without any sex bias (Figure 3A–D, co). In contrast, the high-fat diet induced significant alterations in the systemic immune cell distribution. In both female and male OD mice, the expansion of myeloid cells could be observed (Figure 3A–D, fOD and mOD). Notably, while myeloid cell expansion was associated with a significant reduction in splenic and circulating T cells in male OD mice (Figure 3A,B), female mice revealed preserved T cell numbers (Figure 3C,D). In contrast to myeloid and T cells, no obesity-induced impact on NK cell distribution was observed.

### 2.4. Males Are More Susceptible to Obesity-Induced T Cell Dysregulation than Females

**Figure 4:**
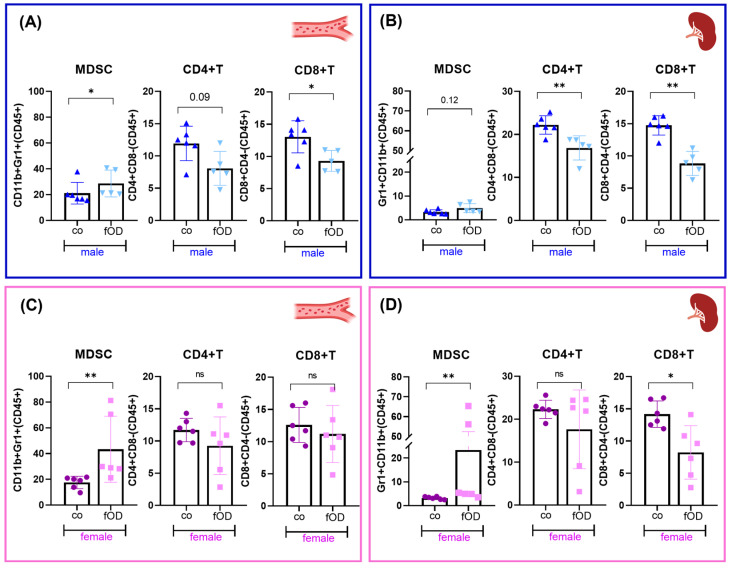
Males are more susceptible to obesity-induced T cell dysregulation than females. Seven-week-old male (blue, **A**,**B**) and female (pink, **C**,**D**) mice were fed standard chow (co) or an obesity-inducing diet (OD). At 45 weeks, blood (**A**,**C**) and spleen (**B**,**D**) immune cells were analyzed by flow cytometry. Cell subtypes are shown as fractions of total CD45+ cells. Results represent the mean ± SD of n = 5–6 mice per group. Differences were tested by Mann–Whitney U test. Significance is indicated for *p* < 0.05 (*) and *p* < 0.01 (**). Non-significant results are labelled ns.

To evaluate sex differences in systemic and splenic immune cell subpopulations in more detail, we next analyzed myeloid and T cell subsets (Figure 4). In both male (Figure 4A,B) and female (Figure 4C,D) mice, the obesity-induced expansion of the splenic and blood myeloid cell fraction mainly consisted of myeloid-derived suppressor cells (MDSCs). While in mOD mice, MDSC expansion in blood (Figure 4A) and spleens (Figure 4B) was associated with a significant reduction in CD4+ and CD8+ T cells, female OD mice revealed preserved numbers of circulating CD4+ and CD8+ T cells (Figure 4C) and splenic CD4+ T cells (Figure 4D) and only slightly decreased splenic CD8+ T cells (Figure 4D).

Taken together, earlier development and progression of MAFLD in male mice was associated with more pronounced reprogramming of systemic and splenic immune cell composition. While obesity-induced expansion of MDSCs was observed in both sexes, male OD mice were more susceptible to the dysregulation of CD4+ and CD8+ T cells. In contrast, female OD mice revealed preserved or only slightly reduced circulating and splenic T cell numbers.

## 3. Discussion

Chronic low-grade inflammation is regarded as a major hallmark for the development of obesity-induced secondary diseases such as MAFLD, T2DM, cardiovascular disease, and several types of cancer [21,22].

Our study is one of few analyzing sex-specific differences in the susceptibility to obesity-induced hepatic and systemic immune dysregulation. We show here that male mice are more susceptible to diet-induced obesity and associated secondary diseases such as fatty liver disease. Accordingly, male mice displayed significantly more pronounced markers of hepatic inflammation than females, which was associated with obesity-induced systemic immune dysregulation including the expansion of MDSCs and concomitant contraction of T cell numbers. In contrast, the female sex was characterized by lower susceptibility to body weight gain and fatty liver disease progression to steatohepatitis. Regarding systemic immune cell alterations, obesity-induced expansion of the myeloid cell compartment of male mice could also be observed in the female mice population. However, in contrast to their male littermates, fOD mice displayed preserved T cell numbers in blood and spleen.

Most preliminary studies investigating obesity-induced immune dysregulation, including those comparing sex differences, have been focused on immune cell alterations in the visceral or subcutaneous adipose tissue [23,24]. Data on obesity-induced systemic immune cell alterations are rare and have been mostly obtained in male mouse models [20]. In line with the results of this study, obesity-induced expansion of MDSCs in male rodents has been reported before [25]. However, to our knowledge, we are the first to evaluate sex differences regarding this innate immune cell population. Obesity-induced accumulation of MDSCs is known to be associated with the development of different cancer entities including hepatocellular [26], oral squamous cell [27] and renal carcinoma [28], pancreatic [29,30], esophageal [31], ovarian [32], and breast cancer [33,34]. Thereby, MDSCs are known to impair T cell activity, which is in line with our findings [33,35]. Interestingly, obesity-induced T cell contraction of both CD4+ and CD8+ T cells was significantly more pronounced in males, whereas female OD mice displayed widely preserved numbers of circulating and splenic T cells. While the obesity-induced reduction in circulating T cells in male rodents and humans has been reported before [12,13], reports on obesity-induced T cell alterations in females are rare. Preliminary studies have shown that in comparison to male littermates, the blood lymphocytes of female mice are more resistant to a high-fat diet [36]. However, to our knowledge, we are the first to describe sex-specific differences in obesity-induced dysregulation of circulating lymphocyte subpopulations, e.g., CD4+ and CD8+ T cells.

Besides sex differences in obesity-induced dysregulation of circulating immune cells, we also observed variances in the splenic immune cell landscape. The spleen is anatomically directly linked to the visceral adipose tissue via the portal vein circulation and a central organ of immune regulation [37]. Thus, over the last few years, the spleen–liver axis has been discussed to play a significant role in modulating obesity-induced inflammation and therefore promoting secondary diseases, especially MAFLD [38,39]. However, studies evaluating splenic immune cell composition in obesity have been rare. Only lately has our group revealed that leptin-deficient obese male mice accumulate splenic MDSCs cells. Furthermore, Tibaes and colleagues reported an obesity-induced reduction in splenic T cell numbers in high-fat diet-fed Wistar rats [40]. Female rats developed a milder obesity phenotype and a preserved splenic cytokine production profile in comparison to male rats; however, no sex-specific difference in splenic T cell numbers was observed [40]. These data are partly in line with the results obtained in the diet-induced obesity mouse model used in this study: We observed the expansion of splenic MDSCs and the concomitant contraction of T cells in male rodents. Among the female OD mice, the numbers of splenic T cells were preserved (CD4+) or only slightly reduced (CD8+).

Taken together, our findings provide novel insights into sex-related differences in obesity-induced hepatic, systemic and splenic immune dysregulation, and the susceptibility to obesity and MAFLD.

Fatty liver disease is one of the main causes of the development of hepatocellular carcinoma (HCC) and liver transplantation [41]. One hallmark of progression from simple metabolic-associated fatty liver disease (MAFLD) over metabolic-associated steatohepatitis (MASH) to liver cirrhosis is intrahepatic inflammation [41,42,43]. Thereby, metabolic dysregulation and aberrant peroxidation of lipid intermediates lead to liver-resident immune cell recruitment and activation resulting in enhanced levels of pro-inflammatory markers including CCL5, IL-1β, and TNF [44], which is in line with our findings. Rising evidence shows that both innate and adaptive immune cell alterations impact the interplay between metabolic and immune dysregulation, dependent on the different stages of fatty liver disease [44]. Regarding the transition from MASH to HCC, the reprogramming of intra-hepatic T cell subpopulations plays a significant role in driving chronic inflammation [45,46,47].

In this study, we observed different sex-dependent obesity-induced impact on circulating T cells. Thereby, preserved blood T cell numbers in female OD mice were associated with significantly less severe stigmata of fatty liver disease and hepatic inflammation. Based on these findings, it could be hypothesized that obese female mice are less susceptible to T cell recruitment from the circulation into the liver resulting in less severe pro-inflammatory hepatic stimuli. However, it must be emphasized that this study has some limitations: Our data are descriptive, and we can only hypothesize rather than confirm a cause–effect relationship between susceptibility to obesity-induced complications and systemic T cell dysregulation. Since estrogen is known to inhibit adipose tissue inflammation leading to improved insulin-induced lipolysis [48], varying hormonal statuses between men and women might explain our observation that female mice are less susceptible than males to obesity. Thereby, distinct sex hormone-driven patterns of fat distribution might result in different body weight phenotypes in males vs. females, as men are more likely to deposit visceral fat than women [49]. Moreover, estrogen has been shown to play an important role in preserving T cell function [48,50,51]. Thus, higher estrogen levels might explain why female mice display preserved circulating and splenic T cell numbers [52,53]. Furthermore, since women experience great hormone fluctuations across their lifespan, differences in circulating sex hormone levels might also account for the inter-individual heterogeneity among females observed in this study. Thus, further studies correlating obesity-induced immunologic alterations with hormonal status are needed.

Besides this, the experimental design (e.g., selection of diet and strain) must be taken into consideration when discussing the limitations of this study. We have used a mouse model fed with a diet combining the atherogenic properties of a cholesterol/cholate mixture and a (not too extreme) high-fat component, which was well accepted by the mice and mimicked the nutrition habits in humans. Diet-induced rodent models are widely used to investigate obesity-induced metabolic and immunologic alterations but the outcomes may depend on the selection of the mouse strain and diet [54]. For example, while Matsuzawa et al. did not observe bodyweight gain in high-fat diet-fed C57BL/6 male mice [55], male mice of the same strain developed a significant obese phenotype when fed with our diet or high-fat/high-sucrose diets of other groups [56]. Varying diet-induced effects in rodents are commonly reported [55,56,57,58,59] and might be caused by different diet compositions, especially the amount of fat content and its impact on flavor and food intake. Distinct susceptibility to obesity-inducing diets impacts not only metabolic but also immunologic dysregulation [56,58]. Especially in terms of diet-induced effects on the T cell response, partly contrasting results have been reported [60,61,62,63,64,65]. In this context, Wang et al. showed that diet-induced T cell dysregulation broadly varies across multiple mouse models [66]. Besides diet- and strain-dependent effects, recent studies indicate that obesity-induced dysregulation of the T cell compartment is also affected by age [67], T cell subsets [68], genetics [69], and—as shown in this study—sex.

In conclusion, the results of this study indicate a significant impact of sex on the development of obesity, as well as the susceptibility to obesity-induced inflammation and obesity-induced secondary diseases. These data are clinically relevant since most nutrition, exercise, and therapeutical recommendations regarding the prevention of obesity and its secondary diseases rely on male study participants. In recent years, the awareness of sex and sex-specific differences in health and disease has been rising, especially regarding non-communicable diseases in industrial countries [70]. In 2021, the European Society of Cardiology (ESC) added the sections “Sex-specific conditions” and “Sex and gender and their impact on health” to their guidelines on cardiovascular disease prevention for the first time [71]. However, the chapters only describe preliminary data about differences and do not take sex differences between women and men into consideration for therapeutic recommendations (yet). In line with this, current WHO fact sheets regarding advice for healthy diet and physical activity, as well as prevention guidelines on other obesity-associated secondary diseases, usually do not differentiate between men and women [72,73]. The results of this study indicate that susceptibility to obesity and associated secondary diseases shows sex-dependent differences. Thus, further research evaluating underlying molecular mechanisms and influencing factors is urgently needed to transfer preclinical findings into clinical use, establish personalized health recommendations, and implement those into prevention guidelines.

## 4. Materials and Methods

### 4.1. Experimental Animal Procedures

C57BL/6 mice were purchased from Charles River Laboratories (Sulzfeld, Germany) and housed in a controlled environment at a pathogen-free facility with a constant temperature (22 °C) under a 12-h light/dark cycle with free access to food and water. At 7 weeks of age, the mice were fed for 38 weeks with standard chow (control) or an obesity-inducing high-fat diet (15% coconut oil, 15% palm oil, 1.25% cholesterol, and 0.5% sodium cholate), with this being a saturated fatty acid-rich adaptation of a previously published atherogenic diet [55]. Both chows were prepared by Ssniff (Soest, Germany). Body weight and food consumption were recorded regularly. At 45 weeks (n = 5–6/group), mice were euthanized by cardiac puncture under ketamine/xylazine anesthesia. Blood samples and liver and spleen tissue were collected and analyzed as described in the following subsections. All animal experimental procedures were approved by the local ethics committee at the University Hospital Regensburg, Germany (No. 54-2532.1-49/13).

### 4.2. Mouse Tissue Processing

Hepatic tissue sections for gene expression were frozen after organ explantation and stored at −80 °C. Liver tissue for histological analysis was fixed for 24 h in a buffered formaldehyde solution (3.7% in PBS) at room temperature, dehydrated by graded ethanol, and embedded in paraffin. After deparaffinization, hematoxylin/eosin staining was performed. Digital images were captured with an Olympus CKX41 microscope equipped with the ALTRA20 Soft Imaging System (Olympus, Hamburg, Germany).

Spleens were minced into pieces in RPMI 1640 supplemented with 10% fetal calf serum (FCS), squeezed through with the plug of a 2 mL syringe, filtered through a 100 µm cell strainer, and centrifuged. Erythrocytes in single-cell suspensions of spleens were lysed with ACK lysis buffer for 3 min at room temperature (RT). For blood samples, erythrocyte lysis with ACK buffer was performed for 5 min at RT after a washing step with flow cytometry buffer (phosphate buffered saline + 2% FCS). All samples were washed with a flow cytometry buffer and passed through a 100 µm cell strainer. Cells were counted with a CASY TT Cell Counter (OLS, Bremen, Germany). 1–3 × 10^6^ cells were used for immunostaining and analyzed using flow cytometry.

### 4.3. Metabolic Serum Parameter

For serum analysis, murine blood was collected by heart puncture under deep anesthesia, clotted (30 min on ice), and centrifuged to remove cellular components. Serum cholesterol and alanine amino transferase (ALAT) levels were measured by commercially available kits (all from Bayer HealthCare, Leverkusen, Germany) according to the manufacturer’s instructions using an ADVIA 1800 analyzer (Siemens Healthcare Diagnostics, Eschborn, Germany) at the Department of Clinical Chemistry and Laboratory medicine (University Hospital Regensburg, Germany).

### 4.4. Hepatic Lipid Content

To quantify hepatic lipid content, total lipids were extracted from liver tissue sections according to the method of Bligh and Dyer with slight modifications [74]. Hepatic triglyceride, free fatty acid (FFA), and cholesterol levels were quantified using the GPO-triglyceride kit (Sigma, Deisenhofen, Germany), the cholesterol/cholesteryl ester quantification kit (BioVision, Mountain View, CA, USA), and the FFAs half micro test (Roche, Mannheim, Germany), respectively, according to the manufacturer’s instructions.

### 4.5. Flow Cytometry

Flow cytometry was performed using a BD FACS Celesta or FACS Fortessa X20. Data were analyzed with the FlowJo software (v10.8.1, v10.9.1, Tree Star, Ashland, OR, USA). The following antibodies were used for immunostaining according to the manufacturer’s instruction: anti-CD45, anti-CD3, anti-CD4, anti-CD8, anti-CD11b, anti-Gr1, and anti-NK1.1 (all BioLegend, San Diego, CA, USA). After staining, cells were incubated for 20 min at 37 °C and 5% CO_2_, washed twice with flow cytometry buffer (PBS + 2% FCS), and subsequently analyzed by FACS. Myeloid cells were defined as CD45+CD11b+, MDSCs as CD45+CD11b+Gr1+ cells, and NK cells as CD45+ NK1.1+CD3−. T cells were defined as CD3+NK1.1.-CD45+, cytotoxic T cells as CD45+ CD3+CD8+CD4− cells, and conventional T helper cells as CD45+CD3+CD4+CD8− cells.

### 4.6. Isolation and Analysis of RNA

For the isolation of RNA, livers were processed as described above. RNA isolation was performed with the RNeasy^®^ mini kit (Qiagen, Hilden, Germany) according to the manufacturer’s instructions. After lysis, RNA was eluted in water and stored at −80 °C. RNA concentrations were measured with the NanoDrop^®^ ND-1000 UV/VIS spectrophotometer (Peqlab, Erlangen, Germany). Reverse transcription was performed with 0.5 µg of RNA in a total volume of 25 µL using the Reverse Transcription System Kit from Promega (Mannheim, Germany). To quantify mRNA expression, a quantitative real-time polymerase chain reaction (qRT-PCR) was performed with the LightCycler II system (Roche Diagnostics, Mannheim, Germany). The results were normalized to the housekeeper gene 18s rRNA and evaluated with the LightCycler software version 3.5 (Roche Diagnostics, Mannheim, Germany) following the manufacturer’s instructions. The qRT-PCR was performed according to the QuantiTect^®^ SYBR^®^ Green PCR Master Mix protocol (Qiagen, Hilden, Germany) using 10 µL of the QuantiTect^®^ SYBR^®^ Green PCR Master Mix, 2 µL of cDNA, and 0.5 µL each of the forward and reverse primers. Primers were synthesized by SIGMA Genosys (Hamburg, Germany) or purchased as QantiTect Primer Assays from Qiagen (Hilden, Germany).

### 4.7. Visualization and Statistical Analysis

Figures were created with GraphPad Prism (v8.0.1, GraphPad Software, La Jolla, CA, USA). The graphical abstract was created with Biorender (https://biorender.com). Statistical analysis was performed using GraphPad Prism (v8.0.1, GraphPad Software, La Jolla, CA, USA). The results represent n = 6 mice per group unless otherwise indicated and are shown as the mean plus the standard deviation. Control and OD mice were compared using the Mann–Whitney U test and significance was indicated for *p* < 0.05 (*) and *p* < 0.01 (**).

## Data Availability

The raw data supporting the conclusions of this article will be made available by the authors upon request.

## Supplementary Materials

The supporting information can be downloaded at: https://www.mdpi.com/article/10.3390/ijms25158234/s1.
