# Supplementary material for: Sex-Dependent T Cell Dysregulation in Mice with Diet-Induced Obesity
Source: Int J Mol Sci. 2024 Jul 28;25(15):8234. doi: 10.3390/ijms25158234 (PMC11311663; doi:10.3390/ijms25158234)
Supplement: Supplementary file 1 [file ijms-25-08234-s001.zip › ijms-3085062-supplementary.pdf]

Supplementary Figure S1

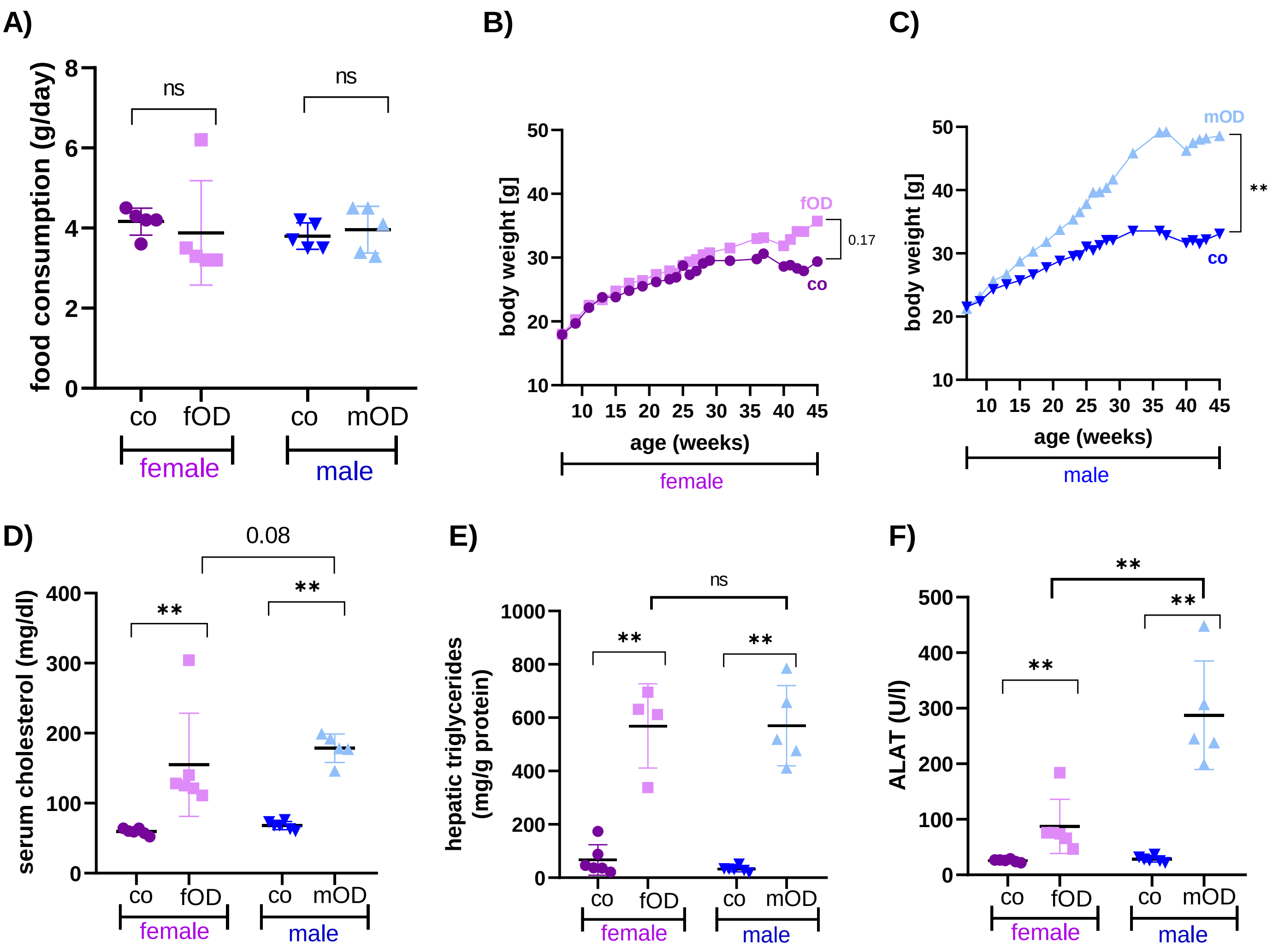

Supplementary Figure S1: Male mice are more susceptible to diet-induced obesity and fatty liver disease than female. (A) Food consumption of female (pink) vs. male (blue) co and OD mice. (B+C) Body weight of female (pink, B) vs. male (blue, C) co vs. OD mice over 45 weeks. (D) Serum cholesterol, (E) Hepatic triglyceride (TG) content and (F) alanine amino transferase (ALAT) at week 45. Results are shown as the mean +/- SD of n=5-6 mice per group. Significance is tested by Mann Whitney U test and indicated for $p < 0.05$ (*), $p < 0.01$ (**), $p < 0.001$ (***).
